# Yearning for machine learning: applications for the classification and characterisation of senescence

**DOI:** 10.1007/s00441-023-03768-4

**Published:** 2023-04-05

**Authors:** Bethany K. Hughes, Ryan Wallis, Cleo L. Bishop

**Affiliations:** Blizard Institute, Barts and The London Faculty of Medicine and Dentistry, Queen Mary University of London, 4 Newark Street, London, E1 2AT UK

**Keywords:** Senescence, Machine learning, Aging, Deep learning, Sc-RNA seq, Artificial intelligence

## Abstract

Senescence is a widely appreciated tumour suppressive mechanism, which acts as a barrier to cancer development by arresting cell cycle progression in response to harmful stimuli. However, senescent cell accumulation becomes deleterious in aging and contributes to a wide range of age-related pathologies. Furthermore, senescence has beneficial roles and is associated with a growing list of normal physiological processes including wound healing and embryonic development. Therefore, the biological role of senescent cells has become increasingly nuanced and complex. The emergence of sophisticated, next-generation profiling technologies, such as single-cell RNA sequencing, has accelerated our understanding of the heterogeneity of senescence, with distinct final cell states emerging within models as well as between cell types and tissues. In order to explore data sets of increasing size and complexity, the senescence field has begun to employ machine learning (ML) methodologies to probe these intricacies. Most notably, ML has been used to aid the classification of cells as senescent, as well as to characterise the final senescence phenotypes. Here, we provide a background to the principles of ML tasks, as well as some of the most commonly used methodologies from both traditional and deep ML. We focus on the application of these within the context of senescence research, by addressing the utility of ML for the analysis of data from different laboratory technologies (microscopy, transcriptomics, proteomics, methylomics), as well as the potential within senolytic drug discovery. Together, we aim to highlight both the progress and potential for the application of ML within senescence research.

## Introduction

### Senescence

Senescence refers to a terminal cell fate defined most notably by the arrest of cellular proliferation (Sharpless and Sherr 2015). Typically, this occurs in response to some form of cellular insult, including exposure to external stressors such as DNA-damaging agents, ionising radiation or hydrogen peroxide (Gorgoulis et al. 2019). Alternatively, the inducting stimuli may manifest as intrinsically hazardous cellular events, such as the activation of oncogenes (Serrano et al. 1997) or the critical shortening of telomeres (Hayflick and Moorhead 1961). Regardless, the initiation of a senescence response is considered a protective mechanism, preventing the propagation of damaged cells and the potential for cancer development (Campisi 2001). However, this protective role against malignancy (sometimes referred to as the “bright-side” of senescence) appears to be balanced against a deleterious role in aging (the “dark-side”), with senescent cells shown to accumulate with age (Campisi et al. 2007; van Deursen 2014). This is attributed to the age-associated impairment of immune function, restricting the elimination of senescent cells (Xue et al. 2007). Thus, as organismal age increases, there appear to be tissue-specific increases in senescent cell accumulation (Tuttle et al*.* 2020). The consequences of this have been strikingly demonstrated through the clearance of senescent cells from naturally aged mice, which prevented the deterioration of key organ systems and led to an extension of median life-span (Baker et al. 2016). This seminal work has subsequently been expanded, with senescent cells demonstrated to contribute to a range of age-related pathologies (Childs et al. 2015; Childs et al. 2016; Sturmlechner et al. 2017). Consequently, there is now widespread ardour towards the potential therapeutic applications of senescent cell clearance, which has led to the development of so called senolytics—agents designed to selectively eliminate senescent cells (Kirkland et al. 2017). However, despite the rapid progress made within the last decade, the senescence field is confronted with a number of fundamental challenges, arising from increasingly distinct senescent contexts, as well as a greater appreciation of the underlying nuances within each setting (Gorgoulis et al. 2019).

### Key challenges in the classification and characterisation of senescent cells

Perhaps the greatest challenge facing the senescence field might be considered fundamental; defining an answer to the question “what is a senescent cell?”. Senescence as a term derives from the Latin *senex* simply meaning “old” and was first used by Peter Medawar to describe the mutation accumulation theory of aging, a concept describing the declining impact of natural selection against harmful mutations expressed after reproduction (Medawar 1952). The term was then used by Hayflick and Moorhead in 1961, to describe the specific cellular property of finite proliferative potential (the Hayflick limit) (Hayflick and Moorhead 1961). Subsequently, a variety of additional terms have emerged including, but not limited to, cell senescence, cellular senescence, replicative senescence, epithelial senescence, immunosenescence and cellular aging (Gems and Kern 2022). This varied nomenclature is indicative of the growing number of biological contexts in which senescent cells have been demonstrated to occur and contribute. Whilst senescence has long been appreciated to occur in response to cell stress or damage, it is now increasingly accepted to function in a number of more homeostatic processes including during wound healing and embryonic development (Muñoz-Espín et al. 2013; Demaria et al. 2014). Furthermore, post-mitotic and terminally differentiated cells are often associated with properties characteristic of senescence due to their non-proliferative state, complicating attempts to distinguish these cell fates (Sapieha and Mallette 2018). Therefore, senescence currently encompasses a variety of disparate processes, and it remains unclear how similar the underlying senescent phenotypes should be considered. An indication that distinct senescence contexts might be more dissimilar than currently appreciated may be found in another of the major challenges within the field, the lack of universally accepted markers of senescence.

As discussed above, senescence has been associated with a range of biological processes, with its function as a cellular stress response perhaps the most well-described. However, even within this context, senescence has been found to be highly heterogeneous, with key sources of variation including the cell type under investigation and the specific stressor used to elicit the senescence response. Furthermore, even within a single experimental model of senescence, individual cells have been demonstrated to reach distinct final phenotypes, through entirely separate mechanisms (Teo et al. 2019). Therefore, in order to aid the identification of senescent cells, the field has developed a series of so-called “hallmarks”, including expression of the cyclin-dependent kinase inhibitors p16 and/or p21 (Alcorta et al. 1996; Espinosa et al. 2003), altered cellular and nuclear morphology (Cristofalo and Kritchevsky 1969; Sadaie et al. 2015), appearance of DNA-damage foci (Fagagna et al. 2003), increased senescence-associated β-galactosidase activity (Dimri et al. 1995), acquisition of a senescence-associated secretory phenotype (SASP) (Coppe et al. 2008), increased secretion of extracellular vesicles (Wallis et al. 2020) and the intrinsic arrest of the cell-cycle (Hayflick and Moorhead 1961). However, aside from the cessation of cellular proliferation, none of these hallmarks are individually present within all senescence contexts and, crucially, are not unique to senescence (Hernandez-Segura et al. 2018). This makes confident identification of senescent cells difficult, an issue exacerbated in vivo, where markers are even more limited. Somewhat circularly, this hinders the comparison of senescent cells involved in the various biological settings described above, given that classification of cells as senescent relies on a set of markers with known limitations (González-Gualda et al. 2021). Therefore, there is currently significant interest in the development of methods to improve both the identification and classification of senescent cells. Furthermore, a greater emphasis is being placed on understanding the heterogeneity of senescence, evidenced by the increasing body of single-cell RNA sequencing (sc-RNAseq) datasets, which allow the characterisation of distinct phenotypes within individual senescence models.

As the field seeks to improve the classification and characterisation of senescent cells, the size and complexity of datasets have inevitably increased. In order to take full advantage of this development, researchers have already begun to apply machine learning (ML) methods to senescent research questions. In this review, we aim to introduce key concepts within the field of ML and outline how these have begun to be utilised within the context of senescence. We aim to particularly focus on how ML has been applied to the tasks of both classifying cells as senescent and characterising the unresolved complexity of senescent cell biology.

## Machine learning

### General principles

**Fig. 1:**
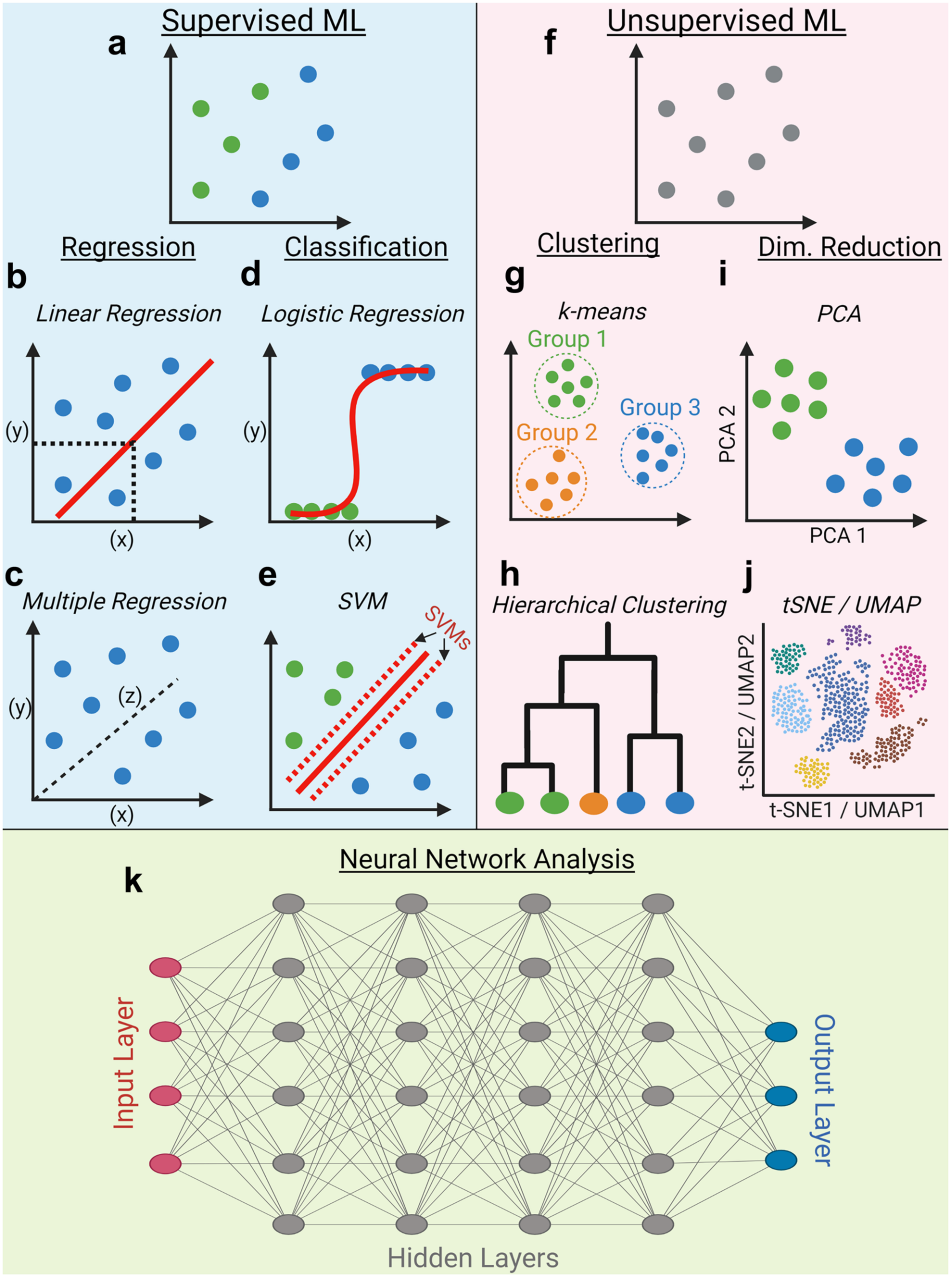
Machine learning methods. Traditional machine learning (ML) methods are classed as either supervised ML (**a**) (where data is labelled with a state of ground-truth) or unsupervised ML (**f**) (where data is unlabelled). Supervised machine learning tasks may employ regression methods, in order to predict numeric outcomes from one or more variables (e.g. linear/multiple regression) (**b**, **c**). Alternatively, classification tasks require the sorting of observations into pre-determined discrete categories (e.g. logistic regression/support vector machines (SVMs)) (**d**, **e**). Unsupervised ML methods are exploratory and may take the form of clustering tasks to determine the similarity between observations (e.g. *k*-means/hierarchical clustering) (**g**, **h**) or dimensionality reduction tasks, which aim to reduce the number of variables under assessment (e.g. PCA and t-SNE/UMAP) (**i**, **j**). Deep learning tasks rely on artificial neural networks (**k**). Variables serve as nodes within an input layer which are connected to an output layer (where model performance is assessed) via a series of connected hidden layers

Arthur Samuel is credited with coining the term “Machine Learning” in 1959, but over 60 years later, there remains no universally accepted definition (Samuel 1959). Broadly, ML may be thought of as the science of programming computers to iteratively improve in some form of data-orientated task based upon experience (Kersting 2018; Sarker 2021). At the base of ML is the concept of an algorithm, which is simply a defined set of instructions that are to be implemented in a particular manner (Flach 2012). ML algorithms generally take input data, perform a specified set of analysis steps and generate an output value. As more data is analysed, the ML algorithm is refined and improved in order to generate a ML model. This model can then be used to analyse new datasets, predicting outcome values based upon what was learned from the original data (Greener et al. 2021; Kingsmore et al. 2021). In general, ML algorithms can be separated into two categories, which are used to perform distinct tasks: supervised learning (Fig. 1a–e) and unsupervised learning (Fig. 1f–j). The distinction between these comes from the input data, with supervised ML applied to labelled datasets where a desired outcome is known, whereas unsupervised learning is exploratory, with the aim to find underlying relationships within unlabelled data (Sommer and Gerlich 2013). In this section, we discuss some of the most widely applied ML algorithms, in order to provide context for those that have been used in senescence research. However, we do not attempt to provide a definitive list or delve into the mathematics behind the algorithms themselves, which has been comprehensively performed elsewhere (Flach 2012; Lantz 2019; Greener et al. 2021; Sarker 2021).

## Supervised learning (regression vs classification)

Supervised learning requires a state of ground-truth within the training data. This takes the form of a value or category associated with an observation, which reflects the reality that the ML model is attempting to learn. Therefore, the labelling of observations within datasets to which supervised ML algorithms are applied is crucial to generating effective models that are able to perform their intended functions (Greener et al. 2021). The tasks most commonly performed via supervised learning methods fall into two categories: regression and classification (Kingsmore et al. 2021; Sarker 2021).

Regression tasks attempt to generate numeric predictions of continuous variables (Stanton 2017). Linear regression is the simplest example of this, where the value of a dependent variable (*y*) can be predicted based upon the value of an independent variable (*x*) (Fig. 1b). More complex multiple regression models can also be built, which generate predictions for the value of the dependent variable based upon multiple independent variables (Fig. 1c). Crucially, the most defining feature of all regression tasks is the generation of a numeric outcome (Lantz 2019).

By contrast, classification ML tasks involve the assignment of observations into discrete predetermined categories (Sommer and Gerlich 2013). One widely used method to achieve this is logistic regression, which makes use of both a binary outcome decision (e.g. “senescent” or “non-senescent”) and a decision boundary in order to classify observations (Fig. 1d). The decision boundary determines the thresholds for classification into one of the two pre-determined groups and is selected by iteratively assessing model performance (see “Performing machine learning: model performance and testing”) (Sarker 2021). Another widely used ML algorithm that has a particular focus on this concept of a decision boundary is support vector machines (SVMs) (Fig. 1e). The SVM is a hyperplane (best thought of as a boundary) that is able to determine the greatest separation between classes of labelled data. This can then be used to predict the membership of new data into each established class (Cortes and Vapnik 1995). Whilst there are numerous other ML algorithms for classification (e.g. Naïve Bayes, decision trees and random forests) all are used to achieve the same fundamental goal, the sorting of new data into pre-established categories (Sarker 2021). In the context of senescence, classification algorithms are most widely implemented with the goal of labelling cells as “senescent” or “non-senescent” (Greener et al. 2021). However, this is hindered by the heterogeneity of senescence and the continued uncertainty regarding the biological definition of senescence, making confident labelling difficult (Hernandez-Segura et al. 2018). Therefore, there is tremendous scope for the second broad category of ML within the senescence field, namely unsupervised learning.

## Unsupervised learning/clustering

Whereas supervised learning requires training data to be carefully labelled, this is not the case with unsupervised learning. Here, the application of ML algorithms is more exploratory, with the aim generally to find unanticipated structure or relationships within the data, thus allowing the characterisation of novel phenotypes (Sommer and Gerlich 2013; Greener et al. 2021). The most common form of unsupervised learning is clustering, which aims to determine the similarities/differences between observations which are then sorted into discrete groups (Fig. 1g) (Kingsmore et al. 2021). Unlike supervised learning, these groups are not predetermined by labelled data, but are sensitive to the context of the ML task, as well as assumptions made by the ML algorithm (so-called hyperparameters) (Greener et al. 2021). The most obvious example of this is the *k*-value used in the widely applied *k*-means clustering ML method. The value of *k* determines the number of groups (known as clusters) that will be output, with data-points which are similar placed into the same group. The selection of the *k*-value dictates the outcome of this clustering, and depending on the specific task to which the algorithm is being applied, several *k*-values may require assessment (Jain 2010). This is the exploratory nature of unsupervised learning, which usually requires iterative testing and refinement to achieve the intended goals (Lantz 2019). A similar and widely applied ML method is hierarchical clustering (Fig. 1h). In this method, Euclidean distances between data points are generally used to assess similarity, with the most similar points combined into a single point, a process that is repeated until all have been linked. This is generally represented by a dendrogram, which allows visualisation of linkage between observations (Gordon 1987).

## Unsupervised learning/dimensionality reduction

Aside from clustering, the most commonly applied methods of unsupervised learning are dimensionality reduction algorithms (Fig. 1i). These aim to take datasets with a large number of variables and distil them down into a smaller, more manageable set, whilst maintaining the relationships within the data as a whole. Examples include principal component analysis, t-distributed Stochastic Neighbour Embedding (t-SNE), and Uniform Manifold Approximation and Projection (UMAP) (Van der Maaten and Hinton 2008; Jolliffe and Cadima 2016; McInnes et al. 2018). These are usually applied to produce more interpretable 2D visualisations of highly dimensional data (Fig. 1j). The specific nuances between these methods have been extensively referenced elsewhere (Kobak and Berens 2019; Do and Canzar 2021).

One limitation of unsupervised learning is that it lacks the state of ground-truth associated with labelled data, making the assessment of model performance more complex. However, this is countered by finding genuinely new information about the relationships within the dataset (see “Performing machine learning: model performance and testing”) (Greener et al. 2021). In senescence research, unsupervised learning has the potential to facilitate the exploration of the heterogeneity of senescence by facilitating the identification and characterisation of novel subgroups within complex datasets.

## Deep learning

Deep learning is a term of distinction from so-called traditional (supervised and unsupervised) ML algorithms (Fig. 1k). At the core of deep learning is the concept of artificial neural networks, which are named for their conceptual similarity to the connections between neurons within the brain (McCulloch and Pitts 1943; Emmert-Streib et al. 2020). Deep learning neural networks comprise a series of layers: an input layer, in which variables from the dataset exist as nodes; a series of hidden layers, with nodes linking to both the previous and subsequent layers; and finally an output layer, from which the results of the analysis are accessible. The neural network learns the relationships and interactions between these nodes, with the complexity increasing with each hidden layer, which can number in the hundreds (Alzubaidi et al. 2021). One of the most popular methods of deep learning is the application of convolutional neural networks (CNNs) which have proved particularly useful in the field of computer vision, as they are able to take an image as an input without the need for complex feature extraction (LeCun et al. 1998; Alzubaidi et al. 2021). Deep learning can be applied to both supervised and unsupervised ML tasks and the mathematics which underpin neural networks have been covered far more comprehensively than is in the scope of this review (Emmert-Streib et al. 2020; Alzubaidi et al. 2021). Therefore, we will focus on the applications of these techniques in the context of senescence.

## Performing machine learning: model performance and testing

The sections above outline some of the most widely applied methods of ML, but they do not begin to scratch the surface of the diversity and complexity of potential ML workflows and applications. Similarly, in this section, we aim to briefly introduce some key concepts for the practical application of ML to data analysis.

When setting out to apply ML to a data set, it is crucial to have a clearly defined task or end-goal against which model performance can be assessed. It is often the case that ML has no “right answer”, particularly in the case of unsupervised learning, where no state of ground-truth exists. In these contexts, iterative and incremental improvements to models can be made, but often a heuristic approach is taken. This is a concept in ML that a model need only be “good enough” to perform its intended task, without necessarily being a perfect solution (Mirshekarian and Sormaz 2018; Greener et al. 2021). The main measure of ML performance is the tangible benefit within its given task (Sommer and Gerlich 2013).

**Fig. 2:**
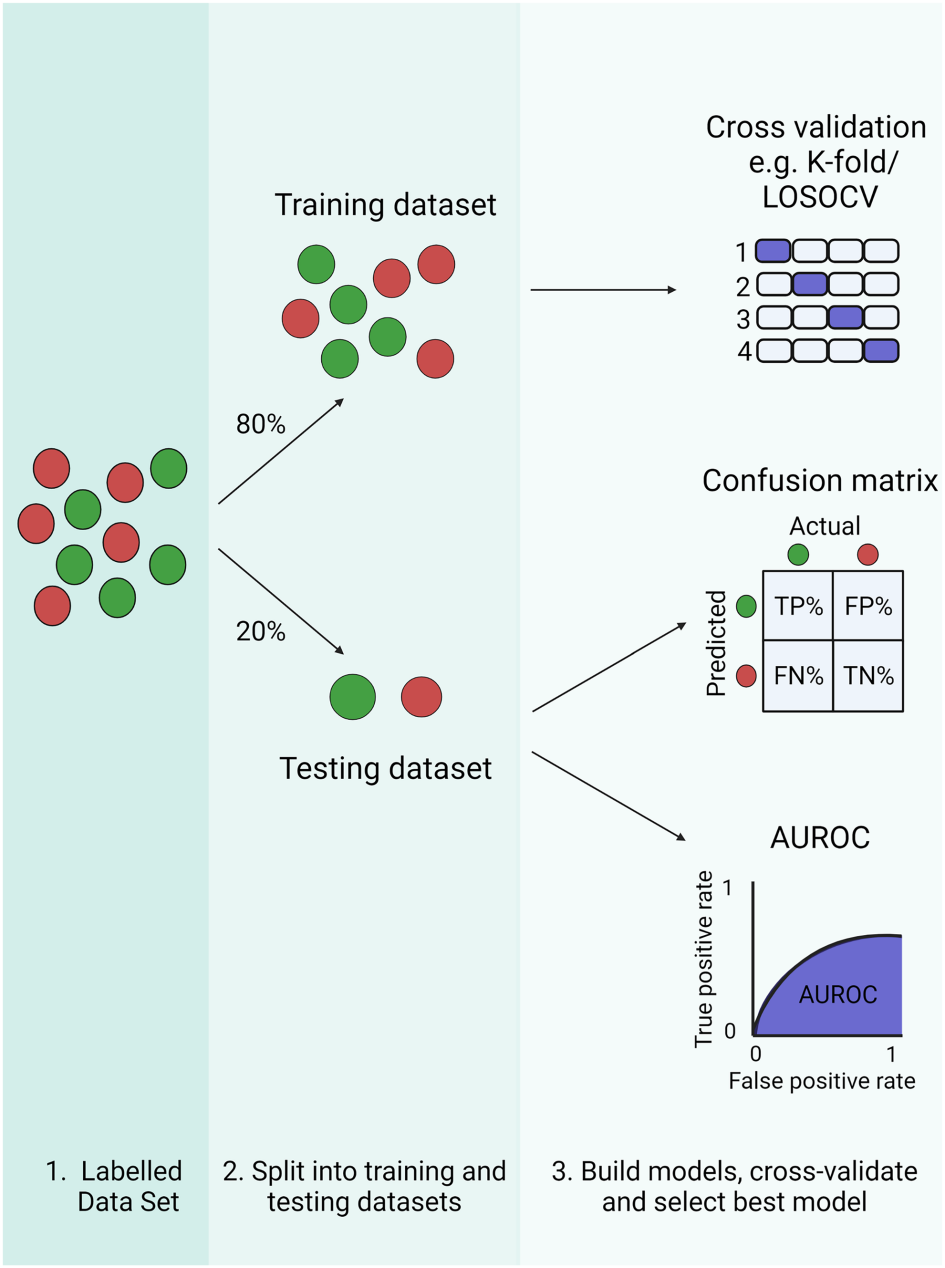
Assessing supervised learning model performance (classification). Supervised machine learning (ML) utilises labelled data sets. In order to construct and assess a classification model, data is divided into training and testing datasets (often 80% training and 20% testing—but this is arbitrary). The training data is used to construct a model through a process of cross-validation, where alternative models are built using different partitions of the training data (e.g. K-fold cross-validation/leave-one-out cross-validation (LOOCV)). The model which performs best on the training data can then be applied to the testing data, in order to assess model performance. Reported metrics include the rate of true/false positives/negatives (often represented in a confusion matrix—TP/FP/TN/FN) and the area under the receiver operating characteristic curve (AUROC), which ranges from 1 (a perfect model) to 0 (a model that makes only false predictions)

Assessing model performance is slightly more straightforward for supervised data (in particular classification tasks), as the labelled training set contains a state of ground-truth (Fig. 2) (Greener et al. 2021). Generally, training data is split into training and testing sub-sets (often 80% training and 20% testing—but this is arbitrary). Methods such as K-fold cross-validation and leave-one-out cross-validation (LOOCV) are used to build multiple models based on different partitions of the training data (Jung and Hu 2015). The main aim is to avoid the issue of overfitting, where a model performs well on training data but poorly on unseen data. Other widely applied methods to minimise overfitting include regularisation methods (ridge, LASSO and elastic nets), which limit the complexity of the model generated, increasing its generalisation to new datasets (Sidey-Gibbons and Sidey-Gibbons 2019). This principle of parsimony (a strong preference towards simple solutions) is prevalent within ML and underpins other methods such as PCA and recursive feature elimination (RFE) (Darst et al. 2018; Vasudevan et al. 2021).

Determining whether a model has made an accurate prediction (one which aligns with the established ground-truth for that observation) is often represented as a confusion matrix, whereby the predicted and actual classifications can be represented and compared, in order to determine the number of false-negatives and false-positives. The performance of classification models may be reported in the form of a receiver operating characteristic (ROC) curve. This is a graphical representation of the relationship between false positive and false negative rates when the decision threshold is varied. By taking the area under the ROC curve (AUROC), an aggregate value of model performance at all possible decision thresholds is produced, with a perfect model producing an AUROC of 1 and model which makes only incorrect predictions producing an AUROC of 0 (Sidey-Gibbons and Sidey-Gibbons 2019). As with unsupervised learning, iterative improvement to workflows is usually required, and the principal measure of a ML method is whether it represents a meaningful improvement over other analysis techniques.

## Classification and characterisation of senescent cell morphologies

High-throughput automated microscopy is a technique that allows the visualisation of large-scale cell screens, usually incorporating a significant number of experimental conditions and stains. However, this leads to the generation of an extensive set of image-stacks, making the manual processing and assessment of images unfeasible (Caicedo et al. 2017). Emerging image-analysis technologies aim to overcome this issue, by providing libraries of defined morphological measurements, which are able to generate extensive textural and contour data of individual cells in an automated fashion. One such example is CellProfiler, an open-source software which provides size, intensity, shape and texture measurements of single cells and nuclei (Stirling et al. 2021). It is important to emphasise that in the context of high-content analysis, these different features are collectively referred to as describing the cell “morphology”, which defines more than the simple geometric shape of the cell (Young et al. 2008). These measurements can range in the hundreds, leading to the generation of large and complex datasets, prime for the implementation of ML workflows (Ljosa et al. 2013). In 2018, CellProfiler introduced the CellProfiler Analyst module, which integrated the phenotypic scoring and ML-based classification of cells within the primary software (Stirling et al. 2021). The development of such user-friendly technologies will continue to accelerate the adoption of ML techniques, increasing the opportunity for the application of ML in new experimental contexts (Chandrasekaran et al. 2020). In the senescence field, this has already begun.

Senescence-associated beta-galactosidase (SA-β-GAL) staining is one of the most widely reported hallmarks of senescence (Dimri et al. 1995). Practically, within the assay, senescent cells acquire a distinctive blue stain of lysosomal compartments, not generally seen in their proliferating counterparts within the same assay window. Cells are often manually assessed using a bright-field microscope and classified as either positively or negatively stained. Chan et al. set out to provide greater nuance to this assay, by utilising high-throughput analysis of SA-β-GAL staining intensity (Chan et al. 2016). The authors conducted a high-throughput siRNA screen in telomerase reverse transcriptase (TERT) immortalised BJ fibroblasts, transduced with myristoylated *AKT1* to produce stable oncogene-induced senescent (OIS) cells. The group screened 10 plates from the siGENOME RNAi library for their ability to induce senescence escape, measured by a reduction in perinuclear SA-β-GAL. A training dataset of approximately 1000 cells was visually classified into 4 groups based on their SA-β-GAL staining: strong, medium, low or none. Supervised random forests were used to build a classification model of the four SA-β-GAL staining groups based on 154 CellProfiler staining intensity morphological features. Subsequently, a single siGENOME RNAi library plate was assessed, in order to determine if any siRNA knockdowns caused senescence escape and whether this could be predicted using the previously constructed random forest model. The group found four siRNA gene targets which appeared to induce a senescence escape as measured by increased cell number. However, these gene targets did not have a significant reduction in the number of cells classified as having strong, medium or weak SA-β-GAL staining based on their staining profiles, suggesting no new senescence escape targets were identified. Nevertheless, this study was a demonstration that SA-β-GAL staining could be feasibly assessed in a high-throughput manner and in combination with sophisticated image analysis software, opening the opportunity for application in other screening contexts (Chan et al. 2016). This also demonstrates the potential value of high-content microscopy for senescence characterisation.

An increase in cell size and alterations in geometric cell shape represent some of the oldest reported hallmarks of senescence (Cristofalo and Kritchevsky 1969; Greenberg et al. 1977). Wallis et al. used high-throughput image analysis to develop a senescence-associated morphological profile, or “SAMP”, for numerous cell types and triggers of senescence (Wallis et al*.* 2022). Using InCarta image analysis software, 62 cell and nuclear image-based features were measured in OIS IMR90s, OIS human mammary epithelial cells (HMECs), replicatively senescent human mammary fibroblasts (HMFs), paracrine senescence from OIS IMR90s and replicative senescence in human dermal fibroblasts (HDFs). Image-based feature profiles of proliferative and senescent cells from each condition were scaled and plotted onto a heatmap. Hierarchical clustering presented two distinct branches of senescent and proliferative cells from all conditions, reinforcing the morphological distinction between these cells. Interestingly, the morphological profiles varied in intensity between different senescence triggers, with the replicative senescent HDFs having the most subtle phenotype. Distinct morphological profiles were also able to be detected in vivo using Palbociclib treated SK-MEL-103 xenografts, with p21 staining used as a senescence classification label. Using a process similar to PCA known as exploratory factor analysis (EFA), the group reduced the dimensionality of the morphology data into eight distinct factors, encompassing size, intensity, spacing and shape. By condensing the 64 measures into eight factors, the distinct separation between proliferative and senescent cells was not lost. However, biological interpretability was considerably improved.

Following the exploration of morphological heterogeneity between different senescence models, Wallis et al. used the single-cell morphological profiles to further explore the intra-model heterogeneity. Using the unsupervised dimensionality reduction techniques t-SNE and UMAP, the group could identify distinct clusters of senescent and proliferative cells within the models. The replicative senescent HDF model showed a less distinct clustering profile between senescent cells and their proliferative counterparts than that seen in the other models, perhaps owing to the more gradual transition into senescence and subsequent heterogeneity of the model (Wallis et al*.* 2022). This exploratory work highlights the utility of ML and high-content analysis to investigate SAMPs and presents an opportunity to build supervised classification models of senescent cells based on their morphological profiles.

Deep learning has also been used to investigate senescent cell morphology changes, and consequently as a classification tool for the prediction of senescent cell burden. Heckenbach et al. used the image-based neural network model Xception to classify control, replicatively senescent (RS), and irradiated (IR) senescent human dermal fibroblast nuclei (Heckenbach et al. 2022). The group achieved 95% accuracy when testing the model within the experiment and 94% accuracy on an independent dataset, suggesting the model was able to intra-and inter-experimentally predict senescence based on nuclear morphology with a high degree of accuracy. To increase confidence in the model, the group compared these predictions to known senescence markers, such as SA-β-GAL, which was found within the perinuclear region of 64.1% of IR and 65.8% of RS nuclei, compared to only 19.6% of controls. Importantly, correlation between predicted senescence and nearby SA-β-GAL staining was reported to be 0.96 for IR and 0.90 for RS cells. There were also high levels of correlation between predicted senescence and p16^Ink4a^, p21^Cip1^ and p53 and a moderate correlation of 0.5 between predicted senescence and the presence of DNA damage markers γH2AX and 53BP1, giving confidence that the neural network model is indeed detecting senescent cells.

To test the adaptability of the model, the group investigated the model’s capability to detect different senescent triggers, cell types and disease states. The model accurately predicted that astrocytes and neurons had a higher probability of being senescent than their proliferative counterparts (7.7% and 9%, respectively), showing that the model was able, at least to some extent, to predict increasing senescence in different cell types. Furthermore, the predictor model trained on IR and RS could also predict increasing senescence probability in progeroid cell lines in vitro, and in the skin, testes and hepatocytes of mice in vivo. To investigate whether the model could also predict different triggers of senescence, the group applied it to human dermal fibroblasts which had been treated with the chemotherapeutic doxorubicin, the electron transport chain inhibitor antimycin A, or the antiretroviral drug atazanavir/ritonavir (ATV/r). Interestingly, the IR and RS models were only able to predict an increased probability of senescence in the doxorubicin-treated fibroblasts, suggesting that these cells may most closely resemble the IR and RS fibroblasts the model was built with. Perhaps further iterations and recursive feature elimination would allow for a more holistic model, particularly with regard to senescence trigger.

Finally, Heckenbach et al. examined the clinical applicability of their model by testing it on 169 human skin samples from the Danish National Register of Pathology. Looking at the predicted senescence scores with age, the group found a significant positive correlation with the RS and IR models, but no age-linked change with new models built for doxorubicin, antimycin A or ATV/r. Unsurprisingly, large variation existed on a donor-to-donor basis. The group investigated whether individual donor senescence predictions could be linked to any health-associated issues and found a significant correlation in the International Classification of Diseases (ICD-10) diagnosis codes for malignant neoplasms and senescence prediction with both the IR and RS models. There was also an indicated but not significant correlation with multiple other known senescence-associated diseases such as osteoporosis, osteoarthritis, and hypertension (Heckenbach et al. 2022). Importantly, this work emphasises the clinical advantage of using ML for biopsy testing and begins to pave the way for ML approaches in predicting senescent cell burden and resultant disease outcomes.

## Transcriptomics

Senescence and its many contexts are associated with differentially expressed genes (DEGs) according to cell type, donor, trigger and temporal kinetics. DNA and RNA sequencing techniques have rapidly advanced in recent years, moving from more traditional selective DNA hybridisations via microarray, to bulk and single-cell whole genome sequencing, all of which have been the focus of efforts to implement ML pipelines.

### Microarrays

Kerber et al. were the first to explore the link between donor age and gene expression profiles using ML, doing so within in vitro lymphoblastoid cell culture lines and blood samples from 104 donors (Kerber et al. 2009). Modelling 2151 constitutively expressed genes via linear regression, the group identified 345 genes (16%) which were significantly altered with age, 48 of these (2.2%) also being associated with donor survival. The gene most strongly associated with age was cell division control protein 42 (CDC42), which has an established role within the control of the cell-cycle and senescence (Ito et al. 2014). Unsurprisingly, when Kerber et al. constructed a LASSO multivariate regression model of biological age versus survival, CDC42 presented the strongest model coefficient (genes most strongly associated with biological age and survival). Using the 13 genes with the top model coefficients, the group generated predictive biological ages for the donors. This significantly correlated with mortality, emphasising the potential utility of biological age predictive models. Kerber et al. also used LASSO regression to directly compare gene expression with survival, which was able to accurately predict mortality even at 10 years post-blood draw, further emphasising the potential of ML classification for predicting human lifespan (Kerber et al. 2009).

Alongside classification, ML has also been used with multiple DNA microarrays to characterise endothelial cell senescence, to identify new pathways and DEGs. Park et al. conducted meta-analysis ML of 8 published senescent endothelial cell microarray datasets (Park and Kim 2021). Using Rank Product, a method which ranks DEGs based on fold-change from the proliferating controls, a total of 648 DEGs were identified across these studies (Breitling et al. 2004; Park and Kim 2021). These DEGs were used to build an Elastic Net model of endothelial cell senescence, with 36 core features. Using LOOCV, the group produced a model with a AUROC score of 0.983, suggesting extremely high levels of sensitivity and specificity when predicting whether their testing dataset cells were senescent or proliferating (Breitling et al. 2004). Using Kyoto Encyclopedia of Genes and Genomes (KEGG) pathway analysis, BioCarta pathways database, and Proportional Integrative Derivative (PID) based principal curves, the group were able to use the Bioconductor package “Pathifier” to quantify pathway abnormalities for proliferative and senescent endothelial cells (Drier et al. 2013; Park and Kim 2021). Once again, the group were able to build an Elastic-net model which consisted of 57 core pathways and an AUROC score of 0.982. The pathway most strongly linked with endothelial cell senescence was “A6B1 and A6B4 integrin signalling pathway”. Interestingly, abnormal integrin signalling and cell adhesion have been linked to senescence (reviewed in (Borghesan and O’Loghlen 2017), including in senescent vascular endothelial cells (Sun et al. 2010). This highlights the importance of ML for reducing measures into a digestible dataset and for the potential identification of new DEGs and pathways involved in senescence.

### Bulk RNA sequencing

Following the emergence of RNA sequencing as a widely available technique to unbiasedly identify DEGs within populations, several groups have utilised the bulk gene signature of senescent cells for both classification and characterisation. Khadirnaikar et al. used *k*-means clustering to classify lung adenocarcinoma into high and low therapy-induced senescence (TIS) subclasses and applied this to predict recovery rates, severity of disease and relapse likelihood (Khadirnaikar et al. 2021). Using WI-38 fibroblasts treated with ionising radiation (IR) or doxorubicin to induce senescence, the group identified 515 common DEGs in the senescence groups compared to their proliferative counterparts. These genes were then used alongside *k*-means clustering to classify RNA sequencing data of lung adenocarcinoma and control samples obtained from The Cancer Genome Atlas. This clustering identified two distinct senescent sample clusters: high senescence (HS), which had a higher expression of senescence-inducing genes than the low senescence (LS) group. The HS group also had a significantly greater mean survival rate than LS, showing the protective role of senescence against lung adenocarcinoma. Unsurprisingly, the two groups also had an altered senescence-associated secretory phenotype (SASP) and an altered epigenetic landscape. Finally, Khadirnaikar et al. used recursive feature elimination to isolate 20 genes that were sufficient to classify lung adenocarcinoma samples as HS or LS. For validation, the group performed *k*-means clustering of an independent cohort of samples and were able to classify these samples as HS and LS, with the HS group still showing a greater survival outcome (Khadirnaikar et al. 2021). This work suggests that the level of senescence in lung adenocarcinoma is a strong predictor of disease outcome and demonstrates a need for ML to allow future disease predictions to be made.

The utility of ML for cancer cell senescence has been further explored by Jochems et al., who have established the classifier “SENCAN” (Jochems et al. 2021). SENCAN works more broadly than the previous study and is built with bulk RNA sequencing data from 13 cell lines with two independent TIS triggers: alisertib (SEN ALI) and etoposide (SEN ETO). The group compared gene expression profiles of these two triggers with their proliferative counterparts, with the aim of building a model which could detect TIS. The majority of DEGs were conserved across both models of senescence suggesting that although the compounds were different, their mode of senescence induction and final senescence phenotype is similar. However, when comparing the DEGs between proliferative and senescent cells treated with alisertib for 7 days versus 14 days (SEN ALI L), the group found over double the number of DEGs with the longer treatment (1275 and 2936, respectively) suggesting that temporal kinetics play a large role in gene expression during senescence and that this may be an important factor to consider when building classification models of TIS. Despite this, the group compared SEN ALI (13 cell lines), SEN ETO (13 cell lines) and SEN ALI L (11 cell lines) with their proliferative counterparts and found 245 genes which had at least twofold change in expression in all cell lines with all triggers.

Next, Jochems et al. explored the use of classification machine-learning methods for TIS, and by LOOCV, discovered that an elastic net classification model using 137 genes could accurately characterise SEN ETO, SEN ALI and SEN ALI L cells as senescent. Importantly, the parental untreated cells within all conditions were not classified as senescent. One cell line could not be correctly classified using the elastic net model, HEP3B with 7-day alisertib treatment; however, these treatment conditions only induced 40–60% senescence. This population heterogeneity presents a potential caveat of using bulk sequencing data with classification models of senescence, as the mean population gene expression will not reflect that a proportion of these cells are senescent. The SENCAN model was also unable to detect senescence in an in vivo xenograft model of non-small cell lung carcinoma, which had been treated with Src homology region 2-containing protein tyrosine phosphatase 2 (SHP2) inhibitor, SHP099, to induce senescence (Jochems et al. 2021). Again, this could be due to tissue heterogeneity and could potentially be overcome by approaches such as single-cell RNA sequencing.

### Single-cell RNA sequencing

Single-cell RNA sequencing (scRNA-seq) is a rapidly growing technique, in which the transcriptome of each individual cell can be sequenced allowing for the detection of sub-populations. This is particularly important in the context of senescence, which is established to be heterogenous. Using ML in this setting would allow the classification of senescence on a single-cell level and would overcome the issues with bulk sequencing described above. Several packages have been designed to enable a streamlined computational method to achieve this, most notably ScPred (Alquicira-Hernandez et al. 2019). ScPred uses all detected gene transcripts within an experiment rather than just DEGS, by first converting genes into principal components to reduce the dimensionality of the data and the computational intensity required. These principal components are then used as attributes to build a ML model, whereby unknown data can be projected onto the principal components to subsequently make classifier predictions.

ScRNA-seq has been used for the characterisation of senescent cell subpopulations, notably to distinguish primary and secondary senescence as two separate genotypes. Teo et al. sequenced OIS H-RasG12V-induced IMR90 cells (ER:IMR90) at several timepoints following senescence induction (Teo et al. 2019). The group used Monocle2 pseudotime trajectory analysis (a method for tracking the change in an individual cell’s progress within a particular process between a start and end state) and found a clear progression towards senescence with time (Trapnell et al. 2014). Interestingly, Teo et al. found that the cells diverged into two distinct endpoints suggesting two different cellular outcomes based on their transcriptome. The group discovered that one endpoint was enriched with a cluster of cells that did not express RasV12, suggesting an alternative route to senescence was taken. To confirm whether this alternative senescence trigger was paracrine, the group co-cultured ER:IMR90 with GFP-IMR90 cells, where the GFP cells underwent secondary senescence (Teo et al. 2019). Single-cell RNA sequencing confirmed that the GFP cells clustered away from the ER:IMR90 cells, suggesting a distinct secondary senescence population with an alternative transcriptome. Importantly, Teo et al. were subsequently able to use Ingenuity Pathway Analysis (IPA) and gene set enrichment analysis (GSEA) to detect Notch signalling as a main regulator of this secondary senescent population (Teo et al. 2019). This suggests that the secondary senescence within OIS models is primarily driven through juxtracrine signalling, rather than the SASP (Hoare et al. 2016). Taken together, this work demonstrates the utility of ML to investigate subpopulations of senescent cells with differing trajectories, which can give an indication as to the senescence trigger.

Classification of senescent cells remains a challenge due to this heterogeneity between trigger type and cell type, which is particularly apparent in vivo. To overcome this, Saul et al. have developed a literature review-based predictive senescence gene set of 125 genes, referred to as “SenMayo” (Saul et al. 2022). Using both bulk and ScRNA-seq datasets of young and older whole bone biopsies, the group confirmed an upregulation of the SenMayo gene set in older humans. This was also apparent in brain biopsies from mice, showing inter-tissue and species applicability of the SenMayo gene set. The SenMayo model could accurately predict a reduction of these senescence-associated genes following senescent cell clearance in *INK-ATTAC* transgenic mice by the drug AP-20187, showing a functional relevance of the model to help the identification of senolysis. This was also apparent in human clinical trials of the senolytics dasatinib and quercetin (D + Q), where RNA sequencing of adipose tissue before and after senolytic treatment showed a significant reduction in SenMayo. To assess the transition between cell states, the group applied Monocle pseudotime trajectory analysis to bone marrow sc-RNAseq data and found that the cells towards the end of the pseudotime scored the most highly with SenMayo, indicating the highest levels of senescence. These cells also coincided with increased *CDKN1A* (p21) and *TP53* expression. Saul et al. characterised these cells by assessing DEGs across the pseudotime trajectory and found an increased expression of macrophage migration inhibitory factor (MIF) and MIF pathway members such as CD74, CXCR4 and CD44. Importantly, MIF expression was increased in bones from the *INK-ATTAK* mice, which was reduced when AP20187 was applied and senolysis occurred (Saul et al. 2022). Together, these data reinforce the link between senescence and aging in vivo. Follow-up studies could involve using ML of RNA sequencing datasets to build classification models of bone-marrow senescence, to determine whether the 125 genes identified in the literature review were also present as classifiers within an unbiased model.

## Proteomics

Whilst transcriptomics is doubtless a powerful tool for characterising senescent cells, it is somewhat limited by the observation that gene expression is not always reflected at the protein level (Lanz et al. 2022). Proteomic changes are of particular note in the context of senescence, as one of the most well-established hallmarks of senescent cells is the acquisition of an enhanced secretory profile of cytokines, chemokines and proteases known as the senescence-associated secretory phenotype (SASP). From its discovery, the SASP has been appreciated to consist of surpassing complexity between different cell types and senescence inducers, further complicated by a dynamic composition across the course of senescence induction. The most comprehensive attempt to profile this heterogeneity has been the development of the SASP-Atlas, which utilised mass spectrometry to assess the secretome from three models of human fibroblast senescence (irradiation, oncogenic-Ras, and cytotoxic compound treatment) and irradiation-induced human epithelial cells (Basisty et al. 2020). Out of 1,091 proteins demonstrated to increase within the fibroblast models, only 150 were seen to do so across all inducers, emphasising the variability of the senescent secretome. In an attempt to find pathways of particular note within these core SASP factors, the authors utilised *k*-means clustering to identify an isolated group of three proteins (CXCL1, MMP1 and STC1). Due to their ubiquity, the authors propose that these could potentially serve as surrogate markers of the SASP, simplifying the required characterisation of the secretome for the purposes of senescence classification. Additionally, STC1 and MMP1 have been previously identified as potential aging biomarkers, strengthening the link between senescent cell accumulation and aging.

Another study utilising the proteomic assessment of SASP factors in biological fluid assessed the concentration of 19 proteins in the serum of 565 cervical squamous cell carcinoma patients by multiplexed ELISA. By utilising multiple linear regression models (constrained through Ridge regression), the authors demonstrated that high expression of specific combinations of SASP components was predictive of poor patient outcomes (quantified by hazard ratio). The authors proposed that these models may represent a useful clinical tool for stratifying patient groups based on prognosis. Importantly, this link was not observed for any SASP factor taken in isolation, emphasising the power of ML to identify useful structure within datasets (Purohit et al. 2020).

Whilst the majority of proteomic studies in senescence tend to focus on the composition of the SASP, there has also been work in defining more general changes in the proteome. Delfarah et al. assessed proteomic changes in replicatively senescence human mammary epithelial cells (HMECs). The authors demonstrated that out of 1209 identified proteins, 45 were upregulated and 27 downregulated. The authors utilised hierarchical clustering and heatmap visualisations in order to demonstrate the consistency between their biological replicates and were able to conclude a clear change in HMEC proteome with replicative exhaustion. Investigators were able to use this distinct proteome to construct a classifier model, termed senescence score, that performed well on an existing transcriptomics data-set, allowing the identification of cells undergoing epithelial senescence with an extremely high degree of accuracy (Delfarah et al. 2021).

## Methylomics

DNA methylation was first associated with the regulation of gene expression by Holliday and Pugh in 1975 and has since been recognised as a vital epigenetic process for the formation of closed heterochromatin and the cessation of DNA transcription (reviewed in (Moore et al. 2013) (Holliday and Pugh 1975). This regulation of the balance of heterochromatic and euchromatic DNA is altered during senescence, with both hypo and hyper-methylation changes observed following methylome array or bisulphite sequencing approaches (Cruickshanks et al. 2013; Lowe et al. 2015; Sakaki et al. 2017). Methylomic changes with senescence have also been quantified through the identification of heterochromatin loss as cytoplasmic chromatin fragments (CCFs) or the formation of senescence-associated heterochromatin foci (SAHFs) to silence key cell-cycle regulators (reviewed in (Crouch et al. 2022)). However, the methylome of senescent cells varies with senescence trigger (Sakaki et al. 2017), again opening the potential for exploratory ML methodologies to aid characterisation.

Many groups have investigated the use of an “epigenetic clock”, that is a measure of the methylation status of DNA, which can predict human biological age. Some of the earliest reported models investigated the change in CpG sites in both blood samples (Hannum et al. 2013) and a multitude of tissues (Horvath 2013) from participants aged 0–101. Unsurprisingly, these predictive models heavily rely on ML techniques, including but not limited to elastic net regression (Hannum et al. 2013; Horvath 2013; Belsky et al. 2022), multivariate linear models (Weidner et al. 2014), stepwise regression (Fan et al. 2021), support vector machines (Fan et al. 2021), random forest regression (Fan et al. 2021) and deep learning techniques (Levy et al. 2020; Galkin et al. 2021; de Lima Camillo et al. 2022). Epigenetic clocks and aging are beyond the scope of this current review; however, these topics have been extensively reviewed in (Field et al. 2018; Zhavoronkov and Mamoshina 2019; Li et al. 2022). Instead, we will focus specifically on instances where ML has been used to characterise and classify senescence.

In a recent preprint, Levine et al. have utilised the methylomic differences of senescent cells to build an elastic-net-based epigenetic biomarker from in vitro samples (Levine et al. 2019). This biomarker, subsequently termed DNAmSen, was trained on mesenchymal stem cells (MSCs) and BJ fibroblasts with replication, OIS, and IR senescence triggers, allowing for a more holistic approach to senescence detection. The DNAmSen predictor, based on 88 CpGs, was subsequently used to detect senescence in vivo, with age correlations of *r* = 0.52 in whole blood, *r* = 0.36 in dermal skin samples and *r* = 0.44 in epidermal skin samples. Importantly, individuals aged 85+ had a significantly higher DNAmSen score, based on their whole blood methylome, than those under 40 years old. DNAmSen was also applicable to age-related disease states such as Werner Syndrome, Hutchinson-Gilford Progeria, chronic-obstructive pulmonary disease (COPD), idiopathic pulmonary fibrosis (IPF) and lung tumours (Levine et al. 2019). This seminal work provides the opportunity for the classification of senescence to aid the monitoring of disease burden, and for further exploration and characterisation of basic senescence biology in a laboratory setting.

Additionally, epigenetic clocks were used to monitor senescence-related disease, namely pregnancy-related pre-eclampsia. Suvakov et al. have previously linked MSC senescence with pre-eclampsia (Suvakov et al. 2019), a pregnancy disorder often characterised by impaired angiogenesis and renal dysfunction. Using the Horvath epigenetic clock (Horvath 2013), the group were able to detect increased epigenetic ages from blood samples during pre-eclamptic pregnancies, but no change in normotensive pregnancies (Suvakov et al. 2021). There was also a significant increase in the estimated age versus actual chronological age with pre-eclampsia compared to normotensive samples. This highlights an opportunity to utilise epigenetic clocks to predict disease burden, based on the predicted age of the patients.

The pre-eclamptic women also had increased blood plasma p16 levels, confirmed also in renal and adipose tissue following C-section, and an abundance in SASP factors such as IL-6 and IL-8. This suggests a potential role for senescence in these pregnancies. To investigate this, Suvakov et al. built an in vitro model of pre-eclampsia, by co-culturing isolated pre-eclamptic MSCs with TNF-alpha-treated HUVECs to mimic the inflammatory environment. The co-culture with pre-eclamptic cells decreased the network length of endothelial cells, mimicking the impaired angiogenesis seen in pre-eclampsia in vivo. When the pre-eclamptic MSCs were treated with the senolytic dasatanib, the network length increased in line with the control normotensive cells (Suvakov et al. 2021). Future work could investigate whether the use of dasatanib in pre-eclampsia could reverse the increased epigenetic age as predicted by the Horvath clock and could also be used for novel pre-eclamptic senolytic and senomorphic drug discovery.

## Drug discovery

The process of drug discovery and development is notoriously lengthy and expensive. Therefore, the pharmaceutical industry is always looking for means to improve efficiency by limiting late-stage failure rates and reducing costs (Vamathevan et al. 2019). Naturally, this has led to the adoption of ML in all phases of the drug development pipeline, with applications including target prediction, compound design and assessment of clinical readouts (reviewed in (Vamathevan et al. 2019)). In the context of senescence, drug development has focused on two main areas: pro-senescence compounds (to impair cellular proliferation in cancer) and senolytics (compounds that selectively eliminate senescent cells), both of which have become the target of ML-based drug discovery efforts.

The earliest work in this area utilised an in silico-based screening approach to identify pro-senescent agonists. Investigators identified potential target proteins with a predicted role in telomere dysfunction, for which existing compound screening data was available. A neural network classifier was constructed based on the structures of 3924 compounds of which 1859 were active against the target panel and 2065 inactive. This model was then used to virtually screen 2,086,587 compound structures, leading to 17,278 potential pro-senescence hits. This was then distilled down to a final set of 147 based on pharmacokinetic and 3D fingerprint prediction. These 147 compounds were then assessed for growth inhibition and senescence induction via MTT and SA-β-gal assays in HCT116 human colorectal carcinoma cells, with CB-20903630 identified as the most potent senescence inducer. Additional validation of the senescence phenotype, including in a 3D spheroid model, confirmed CB-20903630 as a novel pro-senescence compound. This work demonstrates the power of predictive modelling and data mining in silico and the potential of ML to enhance the identification of pharmacological agents in the context of senescence (Bilsland et al. 2015).

As described above, several methods of ML have been used to facilitate the classification of cells as senescent based on morphology alone. Through the use of CNNs, Kusumoto et al. were able to develop a system known as Deep-SesMo, a classification tool which provides a score based upon the probability of a cell being senescent. A major advantage of using CNNs is that they are well suited to image analysis tasks, without the need to define features or identify particular targets within the image. Deep-SesMo utilises phase-contrast input images and makes a prediction of senescence state based upon the cell shape (without the need for additional staining). The CNN model underpinning Deep-SesMo was trained using data from human umbilical vein endothelial cells (HUVECs) induced to senescence via reactive oxygen species, DNA damaging agents or replicative exhaustion. The authors demonstrated that this model also performed well using data produced in different institutions and in another cell type, human diploid fibroblasts. Furthermore, Deep-SeSMo could pick out the specific reduction in senescence cells elicited via treatment with the senolytic navitoclax, within a mixed population of proliferating and senescent HUVECs, suggesting that Deep-SesMo could have utility in identifying novel senolytic compounds. To further demonstrate the potential utility of Deep-SesMo, the authors performed a compound screen using a kinase inhibitor library. Deep-SesMo scores were used to rank compounds, which stratified into those that reinforced the senescence phenotype (high Deep-SesMo scores) and those that suppressed senescence (low Deep-SesMo scores). The four compounds which most potently suppressed Deep-SesMo score (terreic acid, PD-98059, daidzein, and Y-27632·2HCl) were validated to also reduce conventional senescence markers including p16/p21 expression and SA-β-Gal activity. Therefore, Deep-SesMo has the potential to enhance senescence-related drug discovery projects due to the simplicity of its required input data, allowing for high-throughput assessment of compounds (Kusumoto et al. 2021).

## Conclusions and future perspectives

As a nascent field, there remain outstanding questions within senescence research, down to the point of fundamental principles. With an accrescent list of associated biological processes (both deleterious and homeostatic), senescence is increasingly accepted to comprise a series of related, yet distinct terminal cell fates. This heterogeneity is driven by the variety of cell types, tissues, triggers and organisms in which senescence has been studied, leading to the development of senescence “hallmarks” in an attempt to facilitate a degree of standardisation within the field. These hallmarks by definition must be broad, in order to encompass a range of senescence contexts. However, this limits the specificity of individual markers to senescence, with many hallmarks also often occurring during the alternative process of damage, stress and normal cellular function. Consequently, the classification of cells as senescent relies on a set of discrete yet imperfect identifiers. This then hinders the subsequent characterisation of “senescent” cells, as associated phenotypes must always contain a degree of uncertainty, thus undermining the confident identification of senescence-associated profiles.

In our view, this trajectory is only likely to continue, particularly with the launch of initiatives such as the SenNet consortium (www.sennetconsortium.org), which endeavours to generate comprehensive atlases of senescence within a diverse range of tissues, pathologies and ages. Such initiatives will doubtless enhance our understanding of the nuances between senescent cells within these settings, but as datasets continue to grow in size and complexity (particularly with the emergence of multi-omic techniques), it may be that the hallmarks on which the field currently relies become insufficient to capture these intricacies. Fortunately, in the age of ML, this presents an enormous opportunity to employ methods such as unsupervised learning, for the purpose of identifying novel sub-classifications of senescence, which may be defined by a complex relationship between variables that currently eludes the field. This could then facilitate a shift from the current focus of classification methods for defining senescent vs non-senescent cells, to one where specific sub-populations or transitional states could be identified routinely. The development of more sophisticated classification models would be of particular use in the identification of senescent cells in vivo, which remains an unresolved challenge within the field. Furthermore, such models could also help elucidate the relative contribution of senescent cells within specific disease states, potentially allowing the stratification of contexts likely to be susceptible to particular senolytic or senostatic treatments. Probing the complexity of senescence through ML may also enhance the discovery of novel senotherapies, which seem likely to continue to present variable efficacy within different settings. In summary, ML holds enormous promise to enhance the study of senescence, in particular by unveiling the full potential of ever more complex and nuanced datasets, facilitating more elaborate classifications of senescent cells and allowing for more refined characterisation within these settings.
